# Syndromic surveillance of respiratory-tract infections and hand hygiene practice among pilgrims attended Hajj in 2021: a cohort study

**DOI:** 10.1186/s12879-022-07559-0

**Published:** 2022-06-27

**Authors:** Hashim A. Mahdi, Harunor Rashid, Fadi S. Qashqari, Sumyya H. Hariri, Osama A. Marglani, Osamah Barasheed, Aqel Albutti, Ameen S. Alwashmi, Ramon Z. Shaban, Robert Booy, Mohammad Alfelali

**Affiliations:** 1 National Centre for Immunisation Research and Surveillance, The Children’s Hospital at Westmead, Westmead, NSW 2145 Australia; 2 The Children’s Hospital at Westmead Clinical School, Faculty of Medicine and Health, The University of Sydney, Westmead, NSW 2145 Australia; 3 College of Health Sciences, Saudi Electronic University, Jeddah, 23442 Saudi Arabia; 4 Sydney Institute for Infectious Diseases, The University of Sydney, Westmead, NSW 2145 Australia; 5 Microbiology Department, Faculty of Medicine, Umm Al-Qura University, Makkah, 24381 Saudi Arabia; 6 Faculty of Medicine, Umm Al-Qura University, Makkah, 24381 Saudi Arabia; 7 The Executive Administration of Research and Innovation, King Abdullah Medical City in Holy Capital (KAMC-HC), Makkah, 24246 Saudi Arabia; 8 Department of Medical Biotechnology, College of Applied Medical Sciences, Qassim University, Buraydah, 51452 Saudi Arabia; 9 Department of Medical Laboratories, College of Applied Medical Sciences, Qassim University, Buraydah, 51452 Saudi Arabia; 10 New South Wales Biocontainment Centre, New South Wales Ministry of Health, Westmead, NSW 2151 Australia; 11 Faculty of Medicine and Health Susan Wakil School of Nursing, The University of Sydney, Sydney, NSW 2006 Australia; 12 Public Health Unit, Centre for Population Health, Western Sydney Local Health District, North Parramatta, NSW 2151 Australia; 13 Faculty of Medicine in Rabigh, King Abdulaziz University, Jeddah, 22252 Saudi Arabia

**Keywords:** COVID-19, Hajj, Hand hygiene, Influenza-like illnesses, Respiratory-tract infections

## Abstract

**Background:**

The risk of transmission of viral respiratory tract infections (RTIs) is high in mass gatherings including Hajj. This cohort study estimated the incidence of symptomatic RTIs and hand hygiene compliance with its impact among Hajj pilgrims during the COVID-19 pandemic.

**Methods:**

During the week of Hajj rituals in 2021, domestic pilgrims were recruited by phone and asked to complete a baseline questionnaire. Pilgrims were followed up after seven days using a questionnaire about the development of symptoms, and practices of hand hygiene. Syndromic definitions were used to clinically diagnose ‘possible’ influenza-like illnesses (ILI) and COVID-19 infection.

**Results:**

A total of 510 pilgrims aged between 18 and 69 (median of 50) years completed the questionnaire, 280 (54.9%) of whom were female, and all of them (except for one) were vaccinated against COVID-19 with at least one dose. The mean (± SD) of pilgrims’ hand hygiene knowledge score (on a scale of 0 to 6) was 4.15 (± 1.22), and a higher level of knowledge was correlated with a higher frequency of handwashing using soap and water. Among those 445 pilgrims who completed the follow-up form, 21 (4.7%) developed one or more respiratory symptoms, of which sore throat and cough were the commonest (respectively 76.2% and 42.8%); ‘possible ILI’ and ‘possible COVID-19’ were present in 1.1% and 0.9% of pilgrims. Obesity was found to be a significant factor associated with the risk of developing RTIs (odds ratio = 4.45, 95% confidence interval 1.15–17.13).

**Conclusions:**

Hajj pilgrims are still at risk of respiratory infections. Further larger and controlled investigations are needed to assess the efficacy of hand hygiene during Hajj.

## Background

Hajj is an annual Islamic pilgrimage where pilgrims gather in sacred sites in the city of Holy Makkah, Kingdom of Saudi Arabia, to perform certain rituals at specific times. The rites of Hajj include assembly at the Holy Mosque where pilgrims make circumambulations around the monolith called ‘Kaaba’, march between two hillocks and stay in tents at specific locations on the outskirt of Makkah. On designated days of Hajj, all pilgrims stay in tents Mina, a valley at the outskirt of Makkah, when they remain in intense religious contemplation and seclusion (called state of ‘Ihram’) by wearing a pair of unstitched sheets, and by avoiding stitched garbs (including gowns, caps and gloves) and scented toiletries. The inevitable over-crowding with minimal physical distancing between pilgrims amplifies the risks of contracting and disseminating viral respiratory tract infections (RTIs) [1, 2]. Respiratory symptoms are common among Hajj pilgrims, with cough being the predominant symptom reaching as high as 90% among some pilgrims [3]. The rate of influenza-like illnesses (ILI) among Hajj pilgrims varied by nationality of pilgrims and study year [1]. However, the overall prevalence of ILI among pilgrims was high in the past decade; for example, more than 80% among French pilgrims from 2014 to 2017 [4], 63.4% among Australian pilgrims in 2011 [5], and 30.4% among Egyptian pilgrims between 2012 and 2015 [6].

Using hand hygiene intervention properly and frequently is one of the most important infection control and prevention methods against viral RTIs. Only limited studies have explored the practice and effectiveness of hand hygiene intervention among Hajj pilgrims [7–10]. A previous systematic review assessed the practice and effectiveness of hand hygiene among Hajj pilgrims but provided inconsistent and inconclusive evidence on the role of hand hygiene in preventing viral RTIs [7]. A study conducted by our team during the Hajj in 2019 showed that while pilgrims have good knowledge and practice of hand hygiene, only a minority used recommended hand hygiene products and most preferred to use soap and water; overall hand hygiene to prevent contact transmission of RTIs is underutilised [11].

Since the World Health Organization (WHO) declared coronavirus disease-19 (COVID-19) as a global pandemic in March 2020, the government of Saudi Arabia has imposed multiple stringent public health measures and restrictions to curb the spread of COVID-19 during the pilgrimage [2]. Consequently, the number of Hajj pilgrims was considerably downscaled from millions of Muslims globally in normal times to only a few thousand local pilgrims in 2020 and 60,000 pilgrims who were resident in Saudi Arabia at the time of Hajj in 2021 [12].

We are not aware of any study has been conducted to estimate the incidence of RTIs among Hajj pilgrims during the COVID-19 pandemic. Also, pilgrims’ hand hygiene compliance and its effectiveness against RTIs have not been well explored. Therefore, the primary objective of this study was to estimate the incidence of symptomatic viral RTIs among pilgrims who attended the 2021 Hajj season. Additionally, we assessed pilgrims’ hand hygiene behaviour during the pilgrimage and its impact on preventing RTIs.

## Methods

A prospective cohort study was undertaken among Hajj pilgrims in the year 2021. Domestic pilgrims aged ≥ 18 years of both genders who spoke Arabic or English not having any ILI symptoms at the time of recruitment were eligible to take part in this cohort study, while those who reported any ILI symptoms at the time of recruitment or refused to participate were excluded. As a result of COVID-19 restrictions and for the health and safety of pilgrims, the study was conducted distantly via phone calls by the research team. An ethics approval was obtained from the biomedical ethics committee, Umm Al-Qura University, Makkah, Saudi Arabia (Approval no. HAPO-02-K-012-2021-07-708), and other necessary permissions to perform the study were also sought. The informed consent was obtained from all subjects.

The recruitment took place during the week of Hajj rituals, from 8 to 13 of Dhul-Hijjah month of the Arabic calendar corresponding to 19 to 23 July 2021. A convenience sample was drawn from a list of around 1000 pilgrims with their names and contact information, provided by Hajj Hamlahs (tour operators). Among those pilgrims from the list who responded to the phone calls were invited to the study and provided with the study’s objectives and procedures, and those who met the eligibility criteria and gave their consent to participate were asked to be interviewed and complete the baseline questionnaire.

The baseline questionnaire contained questions about participants’ socio-demographic information, presence of pre-existing comorbidities, smoking status, and source of pre-Hajj health information. Pilgrims were asked if they received influenza vaccine within the previous year, and the COVID-19 vaccine before attending Hajj. More specific details related to COVID-19 vaccination were captured such as type/brand of vaccine received, and occurrence of adverse reactions (if any) following vaccination. The final part of the baseline questionnaire captured data related to pilgrims’ knowledge about hand hygiene and perception regarding the effects of different hand hygiene products against RTIs. The questionnaire was devised by using exemplary questions used in a previously published survey [13]. It was then modified based on the 2021 global epidemiological scenario and feedback from leading MG and infectious disease experts.

Seven days after the recruitment, pilgrims who completed the baseline questionnaire were contacted again individually via a phone call to participate in the second phase of the study, which focused on following up pilgrims’ health and hand hygiene behaviour. The follow-up questionnaire contained questions about the presence or absence of constitutional symptoms (subjective fever, fatigue, myalgia, and headache), respiratory symptoms (cough, sore throat, rhinitis, dyspnoea, and smell or taste dysfunction) and gastrointestinal symptoms (diarrhoea, and vomiting). Details about these symptoms including onset date, duration and whether they sought treatment were obtained. Lastly, pilgrims were asked about their hand hygiene behaviour during the Hajj journey; for instance, how frequently they used soap and water, and alcohol-based hand sanitisers to clean their hands, and if they cleaned their hands before or after specific high-risk situations, such as before eating, after toilet action, after nose blowing, sneezing, or coughing and so forth.

This study used a broad definition of a case of RTI; it was defined as the development of one or more respiratory symptoms during the follow-up phase. In order to clinically diagnose a possible case of viral RTIs among our study cohorts, syndromic definitions for ‘possible’ ILI and COVID-19 were applied as per the criteria described and evaluated elsewhere [14, 15]. ILI was defined as ‘a triad of cough, sore throat and subjective fever’ as described by Rashid et al. [14]. The triad had a sensitivity and specificity of respectively 67% and 64% against influenza confirmed by reverse transcriptase-polymerase chain reaction. The COVID-19 infection criterion was defined as the ‘concomitant presence of three or more of the following symptoms: fever, myalgia, cough, and olfactory and taste disorders’ as defined by Fulvio et al. [15], which was found to be highly significantly correlated with a positive molecular diagnosis of COVID-19 (adjusted odds ratio [OR] 18.55, 95% CI 13.77–24.97) and a specificity of 91.2%.

The participant’s responses were directly entered into an online questionnaire through “Microsoft Forms”, a development cloud-based software (Microsoft Office 356, version 2002, Redmond, WA, USA). Subsequently, these responses were exported to a master Excel spreadsheet (Microsoft Office 356, version 2002, Redmond, WA, USA) for data translation, cleaning, and coding before importing into Statistical Package for Social Sciences (SPSS) software (IBM SPSS Statistics for Windows, version 26.0, IBM Corp, Armonk, NY, USA) for analysis. For descriptive analysis, frequencies and percentages were applied to present categorical variables, the incidence of symptoms, and ‘possible ILI’ and ‘possible COVID-19’. The mean (or median with range) ± standard deviation (SD) was used to summarise continuous data. The associations of potential factors with the practices of hand hygiene, and with the estimated incidence of RTIs were analysed using simple logistic regression. The risk estimation was carried out using OR with 95% confidence interval (CI) and a *p* value of ≤ 0.05 was considered statistically significant.

With an estimated incidence of symptomatic RTIs associated with Hajj of 30% in a previous study [6] and an error margin of 5% at a confidence level of 97%, a sample of 400 participants was needed for the study. Considering a high dropout rate, we decided to inflate the sample by 125% and targeted to recruit at least 500 subjects.

## Results

A total of 800 Hajj pilgrims were approached for cohort recruitment, of those 510 (63.75%) agreed to take part in the study and completed the baseline questionnaire. During the second phase, 65 (12.75%) were lost to follow-up (37 did not answer the call and 28 refused to proceed with the study) leaving 445 completing the follow-up.

The participants were aged between 18 and 69 (median of 50) years and 280 (54.9%) were female. Native Saudi pilgrims accounted for 437 (85.7%) of the cohort; further, 276 (54.1%) of the participants held a bachelor’s certificate, 126 (24.7%) were public sector employees and 120 (23.5%) were private sector employees. Of all, 167 (32.7%) reported having one or more pre-existing health conditions, 60 (11.8%) reported being tested positive for COVID-19 before Hajj, and 77 (15.1%) were current smokers. Concerning the vaccinations received by pilgrims before attending the pilgrimage, 297 (58.2%) reported receiving the influenza vaccine, and all of them (except for one pilgrim) were vaccinated with a single dose of COVID-19 vaccine, while 472 (92.5%) received both doses (Table 1).

**Table 1:** Pilgrims’ socio-demographics, medical history and vaccination status (n = 510)

Characteristic	n (%)
Age (years)	
18–25	55 (10.8)
26–39	109 (21.3)
40–55	209 (41)
56–64	127 (24.9)
≥ 65	10 (2)
Gender	
Male: female	230: 280 (45:55)
Nationality	
Saudi Arabia	437 (85.7)
Egypt	28 (5.5)
Yemen	8 (1.6)
Tunis	4 (0.8)
Syria	4 (0.8)
Sudan	4 (0.8)
Others	25 (4.8)
Education	
No education	9 (1.8)
Primary/elementary school certificate	20 (3.9)
High school certificate	64 (12.5)
Diploma	27 (5.3)
Bachelor’s certificate	276 (54.1)
Higher university degree	114 (22.4)
Occupation	
Unemployed	107 (21.0)
Student	31 (6.1)
Retired	94 (18.4)
Public sector worker	126 (24.7)
Private sector worker	120 (23.5)
Self-employed	32 (6.3)
Presence of chronic medical conditions	
Hypertension	66 (12.9)
Diabetes	46 (9.0)
Obesity	19 (3.7)
Chronic lung disease	19 (3.7)
Chronic heart disease	10 (2.0)
Smoking status	
Non-smoker	375 (73.5)
Current smoker	77 (15.1)
Past smoker	37 (7.3)
Passive smoker	21 (4.1)
Received influenza (flu) vaccine	
No	213 (41.8)
Yes	297 (58.2)
Received first dose of COVID-19 vaccine	
Oxford/AstraZeneca	98 (19.2)
Pfizer	410 (80.4)
Moderna	1 (0.2)
Received second dose of COVID-19 vaccine	
Oxford/AstraZeneca	38 (7.5)
Pfizer	433 (84.8)
Moderna	1 (0.2)
Reported COVID-19 vaccine adverse reactions	
Pain/redness at injection site	149 (29.2)
Fatigue and headache	153 (30)
Muscle pain	123 (24.1)
Fever/chills	116 (22.7)
Difficulty in breathing	5 (1)
Nausea/vomiting	5 (1)
Diarrhoea	7 (1.4)

The denominator is the number of recruited pilgrims in the study irrespective of whether they completed the follow-up or not

Pilgrims sought some form of health information on preventative methods from various sources: 319 (62.5%) acquired information from social media and internet websites, 283 (55.5%) from the Saudi Ministry of Health campaigns, 205 (40.2%) from Hajj tour operators, 152 (29.8%) from friends and relatives and 82 (16.1%) from health professionals. The mean (± SD) of total knowledge scores (on a scale of 0–6) was 4.15 (± 1.22), nearly half (49%) had a medium score of 3–4, another 41% had a high score of 5–6 and a small proportion (10%) of the pilgrims had a poor knowledge score of 1–2. Most pilgrims (416, 81.6%) believed that soap and water were very effective hand hygiene methods against RTIs, and many others (308, 60.4%) believed alcohol-based hand sanitiser was so effective.

The pilgrims’ hand hygiene practices during the Hajj in the follow-up phase illustrated that 311 (70%) reported washing their hands with soap and water more than five times per day, whereas just over half of them (236, 53%) reported using alcohol-based hand sanitisers more than five times a day. Compared to those with a poor knowledge level about hand hygiene, pilgrims with higher knowledge level had a higher frequency of washing hands using soap and water (77.3% [140/181] vs. 61.5% [24/39], *p* = 0.02). Most pilgrims cleaned their hands after toilet action (417, 93.7%) followed by hand washing before eating (405, 91%) and after eating (391, 88%) (Table 2).

The main reported motivators to comply with regular hand hygiene during Hajj were a strong belief that cleansing hands would protect from infections (281, 63.1%), and an awareness of the protective role of regular hand hygiene practice (326, 73.3%). In contrast, the barriers to compliance with hand hygiene were failure to remember or being too busy to clean their hands (80, 18%) and avoiding scented or alcoholic hands cleaning products (48, 10.8%)

The incidence of RTIs was 4.7% (21 of 445) among pilgrims who self-reported developing one or more symptoms of RTIs during follow-up; of 21 symptomatic pilgrims, 16 (76.2%) had a sore throat, nine (42.8%) had cough, eight (38%) had rhinitis, four (19%) had smell or taste dysfunction, and three (14.2%) had dyspnoea (Table 3). The onset date of symptoms ranged from 23 to 31 July 2021 (i.e., from the last day to one week after the conclusion of Hajj rituals), and the duration was between one to seven (median of five) days. Only one-third of those cases (7 of 21) had visited a hospital or clinic to treat the symptoms. The estimated incidence of ‘possible ILI’ was 1.1% (5 of 445), and the incidence of ‘possible COVID-19’ infection was 0.9% (4 of 445)

**Table 2:** Pilgrims’ hand hygiene practices during Hajj (n = 445)

Hand hygiene practices	n (%)
Frequency of washing hands using soap and water	
Less frequently (≤ 5 times/day)	134 (30)
More frequently (> 5 times/day)	311 (70)
Frequency of using alcohol-based hand sanitisers	
Less frequently (≤ 5 times/day)	209 (47)
More frequently (> 5 times/day)	236 (53)
Practising hand hygiene at key actions	
Before eating	405 (91)
After eating	391 (88)
After toileting	417 (93.7)
When hands are visibly dirty	331 (74.4)
After nose-blowing, sneezing or coughing	254 (57.1)
After touching garbage	276 (62)
After shaking hands	236 (53)
After touching frequently used surfaces	352 (79.1)

The denominator is the number of pilgrims who completed the follow-up

**Table 3:** Reported clinical symptoms of RTIs among pilgrims (n = 21)

Reported symptoms	n (%)
Sore throat	16 (76.2)
Cough	9 (42.8)
Fatigue	9 (42.8)
Rhinitis	8 (38)
Subjective fever	7 (33.3)
Myalgia	5 (23.8)
Headache	4 (19)
Smell/taste dysfunction	4 (19)
Diarrhoea	4 (19)
Short of breath (Dyspnoea)	3 (14.2)

Table 4 illustrates the relationship of potential factors with the incidence of RTIs among pilgrims using simple logistic regression analysis. The analyses showed that apart from obesity, no other socio-demographic characteristics were significantly associated with the risk of RTIs. Being obese significantly increased the increased risk of RTIs (OR 4.45, 95% CI 1.15–17.13, *p* = 0.03). Neither washing hands using soap and water (OR 0.71, 95% CI 0.25–1.99) nor using alcohol-based hand sanitisers (OR 1.02, 95% CI 0.42–2.47) had a significant impact on reducing the risk of developing RTIs. Practising hand hygiene when hands are visibly dirty, after shaking hands, and after touching frequently used surfaces or objects were not significantly protective. Receipt of influenza vaccine tended to be protective against ILI although the difference was not statistically significant (OR 5.77, 95% CI 0.64–52.12, *p* = 0.07).

**Table 4:** Potential factors associated with RTIs among pilgrims (n = 21)

Variable	Number of cases (%)	OR (95% CI)	*p*-value
Age (years)			
18–25	1 (2.1)	Reference	0.37
26–39	8 (8.2)	4.18 (0.50–34.41)	
40–55	6 (3.4)	1.68 (0.19–14.29)	
56–64	5 (4.3)	2.13 (0.24–18.78)	
≥ 65	1 (10)	5.22 (0.29–91.37)	
Gender			
Male	10 (5)	Reference	0.80
Female	11 (4.5)	0.89 (0.37–2.15)	
Nationality			
Saudi	18 (4.7)	Reference	0.91
Non-Saudi	3 (5)	1.07 (0.30–3.76)	
Education level			
No education	0 (0)	0.00 (0.00–0.00)	0.82
Primary/elementary school certificate	0 (0)	0.00 (0.00–0.00)	
High school certificate	4 (7.4)	1.73 (0.55–5.43)	
Diploma	2 (8.7)	2.06 (0.44–9.62)	
University degree	15 (4.4)	Reference	
Chronic medical conditions			
Obesity	3 (16.7)	4.45 (1.15–17.13)	**0.03**
Smoking Status			
Non-smoker	18 (4.8)	Reference	0.91
Current smoker	3 (4.5)	0.94 (0.26–3.27)	
Using soap and water			
Less frequently (≤ 5 times/day)	5 (3.7)	0.71 (0.25–1.99)	0.52
More frequently (> 5 times/day)	16 (5.1)	Reference	
Using Alcohol-based hand sanitisers			
Less frequently (≤ 5 times/day)	10 (4.8)	1.02 (0.42–2.47)	0.95
More frequently (> 5 times/day)	11 (4.7)	Reference	
Hand hygiene practices in key occasions*			
Before eating	18 (4.4)	2.45 (0.56–10.63)	0.23
After eating	18 (4.6)	1.48 (0.34–6.33)	0.59
After toileting	21 (5)	0.00 (0.00–0.00)	0.99
When hands are visibly dirty	17 (5.1)	0.52 (0.14–1.95)	0.33
After nose-blowing, sneezing or coughing	11 (4.3)	1.42 (0.42–4.81)	0.57
After touching garbage	12 (4.3)	1.58 (0.45–5.53)	0.47
After shaking hands	12 (5.1)	0.61 (0.20–1.90)	0.40
After touching frequently used surfaces	17 (4.8)	0.86 (0.25–2.95)	0.81

Significant results are highlighted in bold *Practising hand hygiene in key occasions set as a comparison reference

## Discussion

This appears to be the first study that estimated the incidence of symptomatic RTIs among Hajj pilgrims amidst the ongoing global COVID-19 pandemic, which found that 4.7% of the total pilgrims who completed the study reported having one or more respiratory symptoms, and ‘possible’ ILI and COVID-19 cases were identified in 1.1% and 0.9% of pilgrims, respectively. Sore throat and cough were the most common clinical manifestations presented in this study, and this was similar to what other studies recorded among pilgrims from Australia [5, 8, 16], France [4, 17, 18], and Malaysia [19] who attended Hajj years prior to the pandemic. On the contrary, compared to what was found in our study, the previous studies documented relatively higher rates of ILI ranging from 10% among Australian pilgrims in 2015 [8] to 47.3% among French pilgrims in 2013 [18]. The estimated incidence of ‘possible COVID-19’ among Hajj pilgrims in the current study of 0.9% indicates religious MGs carry an important risk of pandemic despite the high vaccination rate. However, this rate was much lower when compared with the Saudi Arabian nationwide data. A serosurveillance study showed that the overall prevalence of COVID-19 among the Saudi population to be about 11% [20], and another study involving Saudi healthcare workers showed that 10% of them had the disease [21]. A high vaccination rate among pilgrims may have made the difference. During the influenza pandemic of 2009, a prospective survey of 551 vaccinated Egyptian pilgrims reported no influenza A(H1N1)pdm09 among returned travellers. Without vaccination, a much higher incidence would be expected [22]. Very large outbreaks of COVID-19 occurred in religious MGs where vaccination was not mandatory. During the Kumbh Mela (Hindu religious MG) in India in April 2021 a large super-spreader event accelerated waves of COVID-19 throughout India spread via unvaccinated pilgrims [23].

The Saudi Government’s preparedness and response for controlling the COVID-19 outbreak at the recent two Hajj years undoubtedly played a crucial role in controlling the RTIs among pilgrims. For the Hajj 2021, only 60 thousand pre-selected Saudi Arabian residents were permitted to take part, while overseas pilgrims, those with chronic diseases, children (less than 18 years) or elderlies (above 65 years) were not allowed [9]. Besides, the Saudi health authority implemented a bundle of control measures that included pharmaceutical and non-pharmaceutical interventions, mass testing and triaging. In addition, pilgrims’ adherence to preventative measures including maintaining physical distancing and wearing face masks, was monitored stringently [24].

Obesity was identified to be a significant risk factor in this study, and obese pilgrims had a more than four-fold increased risk of RTIs. Review studies that explored the relationship between obesity and viral RTIs concluded that obesity raises the risk of contracting and having severe complications from ILI and COVID-19 [25–27]. Another study that investigated the correlation between the prevalence of obesity and COVID-19 infection rate in 54 countries detected that the residents of Saudi Arabia have the highest prevalence of obesity of 35% and it was significantly associated with the susceptibility to COVID-19 [28]. Furthermore, the increased body mass index (BMI) was observed to have a strong association with hospital admission among patients with COVID-19 infection [29, 30].

Nearly half of the pilgrims in this cohort achieved a moderate level of hand hygiene knowledge, and the mean score on a scale of 0 to 6 was 4.15 (± 1.22). Although the awareness level was expected to improve significantly in this study compared to previous years as a result of the intensive public health campaigns about COVID-19, similar moderate levels of awareness were observed in our formerly conducted surveys involving domestic Hajj pilgrims in 2019 and visitors to the Prophet’s Mosque in 2020, the average scores in a scale of 0 to 12 were 6.7 and 6.4, respectively [7, 13]. More pilgrims in the current study agreed that hand hygiene methods are essential for protection against viral RTIs in comparison to the visitors to the Prophet’s Mosque in 2020: 81.6% of pilgrims believed that the use of soap and water was a very effective method to ward off RTIs while 60.4% pilgrims believed alcohol-based hand sanitiser was effective, these compare with respectively 48.8% and 56% visitors to the Prophet’s Mosque believing the effectiveness of the measures [13]. This may indicate that more pilgrims have now become aware of the importance of hand hygiene possibly an effect of the widespread health education campaigns against COVID-19.

In line with the finding of this study, Alqahtani et al. [8, 31] revealed that a strong belief in the effectiveness of hand hygiene in infection prevention was among the main reasons influencing pilgrims’ decision to use hand hygiene regularly during Hajj. Conversely, obstacles to adherence to hand hygiene during Hajj were also identified in the study, primarily were failure to remember or being too busy and avoiding scented and alcoholic hygienic products. This was an expected result since pilgrims spend a significant amount of time performing rituals and moving between sites during the pilgrimage. Furthermore, according to the rules of ‘Ihram’, scented toiletries including scented soaps, perfumes, or deodorant on pilgrim’s body or clothing are avoided. The religious and cultural preference of Muslims to avoid products that contain alcohol when alternatives are available may further explain the low compliance of using alcohol-based hand sanitiser among Hajj pilgrims [27]. Unlike the reported barrier in our 2019 Hajj study [7], the unavailability of soap and hand sanitisers was not a major hindrance for pilgrims who attended this Hajj to clean their hands, and this was possibly due to the distribution of a large volume of hygienic products at every Hajj sites.

Our results demonstrated that pilgrims who scored higher knowledge of hand hygiene were more likely to wash their hands more frequently using soap and water during Hajj rituals; however, previous studies have not reported a similar correlation between knowledge and practice of hand hygiene among Hajj pilgrims. It has been evident that delivering pre-Hajj health education is an effective method to improve pilgrims’ practices of hand hygiene during the Hajj journey and therefore health promotion and education on hand hygiene should be sustained for Hajj pilgrims [31].

There is still a paucity of data on the effectiveness of hand hygiene practices in preventing RTIs during this Islamic pilgrimage. In this observational study, hand hygiene was not found to be significantly effective against viral RTIs, as was found in a few previous studies involving Hajj pilgrims [9]. In contrast, other studies conducted among US pilgrims in 2009 and Malaysia pilgrims in 2013 confirmed that good hand hygiene was significantly associated with a decreased risk of RTIs [14, 32]. Using a compulsory set of pharmaceutical and non-pharmaceutical interventions during this Hajj may have confounded the real effect of hand hygiene in this study. A randomised controlled trial (RCT) is needed to quantify the role of hand hygiene against RTIs.

This study has added valuable evidence that may be useful for future studies to build, especially the provision of syndromic data for the surveillance of RTIs in the context of a pandemic at such MGs. However, undertaking this study amidst such extraordinary conditions was fraught with some challenges and limitations. We were compelled to investigate only domestic pilgrims from selected Hamlahs which may have restricted the generalisability of the results outside the domain of the defined time and population. Furthermore, we were unable to collect biological samples for virological confirmations due to administrative restrictions and therefore the cases of influenza and COVID-19 were considered possible rather than confirmed. The design of the study was anecdotal in nature; thus, some data could have been at risk of information and recall bias. Other measures in the bundle of infection control such as use of face mask, other personal protective equipment (PPE) and cough etiquette were not explored in this study for the following reasons: first, face mask has been extensively studied at Hajj including through a large RCT [33]; second, facemask use was made compulsory during the Hajj 2021, a penalty was applied for non-compliance; third, it was deemed unlikely pilgrims would use other PPEs (gowns, gloves and eye shields) as they avoid using certain garbs in the state of ‘Ihram’; fourth, cough etiquette is a multi-component prevention strategy having three or more elements in it of which this study already covers one component (hand hygiene practice after coughing, nose-blowing and sneezing). Future studies should explore all components of cough etiquette and possibly also explore PPE use.

## Conclusions

The incidence of syndromic viral RTIs among Hajj pilgrims during the COVID-19 pandemic was low. Stringent preventive measures against COVID-19 may have contributed to reduced incidence. Even though the findings of this study did not show a substantial improvement in hand hygiene knowledge among the pilgrims, a higher level of knowledge did improve hand hygiene practice. Our results could not confirm the protective role of hand hygiene against RTIs during Hajj, it is strongly advised to continue to use hand hygiene and health measures to prevent RTIs at MGs, particularly in the context of a pandemic with a highly transmissible variant like Omicron strain of SARS-CoV-2. An RCT study to explore the effect of hand hygiene against RTIs at Hajj is a call of the time.

## Data Availability

The datasets generated and/or analysed during the current study are not publicly available due to containing information that could compromise participants’ privacy but are available from the corresponding author HR on reasonable request.

## Abbreviations

RTI: Respiratory-tract infection
ILI: Influenza-like illness
MG: Mass gathering
COVID-19: Coronavirus disease-19
